# Encoding Temporal Regularities and Information Copying in Hippocampal Circuits

**DOI:** 10.1038/s41598-019-55395-1

**Published:** 2019-12-13

**Authors:** Terri P. Roberts, Felix B. Kern, Chrisantha Fernando, Eörs Szathmáry, Phil Husbands, Andrew O. Philippides, Kevin Staras

**Affiliations:** 1 Sussex Neuroscience, University of Sussex, Brighton, BN1 9QG UK; 2 Centre for Computational Neuroscience and Robotics, School of Engineering and Informatics, University of Sussex, Brighton, BN1 9QJ UK; 3 School of EECS, Queen Mary University of London, E1 4NS London, UK; 4 Present Address: Google DeepMind, London, N1C 4AG UK; 5 Parmenides Center for the Conceptual Foundations of Science, 82049 Pullach, Munich Germany; 6 Institute of Evolution, Centre for Ecological Research, 3 Klebelsberg Kuno Street, 8237 Tihany, Hungary

**Keywords:** Electrophysiology, Data processing, Hippocampus

## Abstract

Discriminating, extracting and encoding temporal regularities is a critical requirement in the brain, relevant to sensory-motor processing and learning. However, the cellular mechanisms responsible remain enigmatic; for example, whether such abilities require specific, elaborately organized neural networks or arise from more fundamental, inherent properties of neurons. Here, using multi-electrode array technology, and focusing on interval learning, we demonstrate that sparse reconstituted rat hippocampal neural circuits are intrinsically capable of encoding and storing sub-second-order time intervals for over an hour timescale, represented in changes in the spatial-temporal architecture of firing relationships among populations of neurons. This learning is accompanied by increases in mutual information and transfer entropy, formal measures related to information storage and flow. Moreover, temporal relationships derived from previously trained circuits can act as templates for copying intervals into untrained networks, suggesting the possibility of circuit-to-circuit information transfer. Our findings illustrate that dynamic encoding and stable copying of temporal relationships are fundamental properties of simple *in vitro* networks, with general significance for understanding elemental principles of information processing, storage and replication.

## Introduction

The ability of the nervous system to extract and exploit temporal regularities from the sensorium, and to generate and manipulate internal signals with complex temporal characteristics, are fundamental properties that underpin much of the information processing inherent in the production of adaptive behaviour^1^. In general, neuronal circuits have a remarkable capacity to reproduce spatiotemporal dynamics of the inputs to which they are exposed^2,3^ and recent work suggests that simple circuits might have an intrinsic tendency to encode sequential input patterns that act as stimuli^4^. Here, we test this idea directly, focusing on reproducing interval timings as a representative example of the extraction of temporal regularities. We chose this paradigm, not only because it is readily amenable to experimental investigation, but because timing is a critical feature of information processing in the nervous system, relevant to sensory discrimination tasks, motor coordination and learning^5^. Nonetheless, the fundamental neuronal mechanisms responsible for encoding event durations and intervals remain largely elusive^2^. Some dedicated models propose that temporal discrimination relies on referencing specialized neuronal oscillators that give a linear metric of time^6,7^, but recent evidence suggests that interval encoding and pattern recognition can also be an intrinsic feature of cortical circuits where the temporal architecture of the network becomes shaped by the timing of inputs received^2,3,8^. It is not clear, however, whether this is an emergent property of complex interactions between elaborate and natively-wired circuits, or alternatively reflects an inherent interval encoding capability at the level of individual neurons, based on the use of more general intrinsic abilities to extract temporal regularities. Elegant studies using dissociated neuronal cultures hints at the intrinsic nature of such computations^9^, suggesting that reduced circuits can perform simple forms of pattern recognition^10,11^. A related question is whether the temporal restructuring of firing relationships could also provide a template for replication, allowing stored changes in the temporal processing architecture of one circuit to be transferred to other independent networks, thus enabling replication of temporal relationships and temporal processing within a neural substrate. The idea of an information copying capability in the brain is of significant interest^12^, particularly for learning and memory circuits, but has yet to be directly demonstrated.

Here we investigate these questions using sparse reconstituted hippocampal circuits and multi-electrode array (MEA) technology. Cross-correlation analysis demonstrates that such simple networks can be trained to encode an imposed 50–200 millisecond-order temporal interval between two neuronal populations (A and B), stored in the temporal relationships of their spontaneous firing activity and persisting over an hour timescale. A trace of the same interval relationship can be recorded in unstimulated regions that act as information conduits between the trained neuronal populations. We show that this learning is accompanied by positive increases in mutual information between activity in A and B, and transfer entropy from A to B, formal measures related to information storage and flow respectively. We also show that the output relationships derived from trained circuits can provide substrates for replicating interval patterns in other naïve networks, facilitating network-to-network information transfer. Our results suggest that the adaptive storage of temporal information and its stable translation to other discrete circuits are intrinsic properties of even simple networks, with general significance for understanding fundamental principles of information encoding and replication.

## Robust Learning of Temporal Relationships in a Simple Network

### Materials and methods

**Figure 1:**
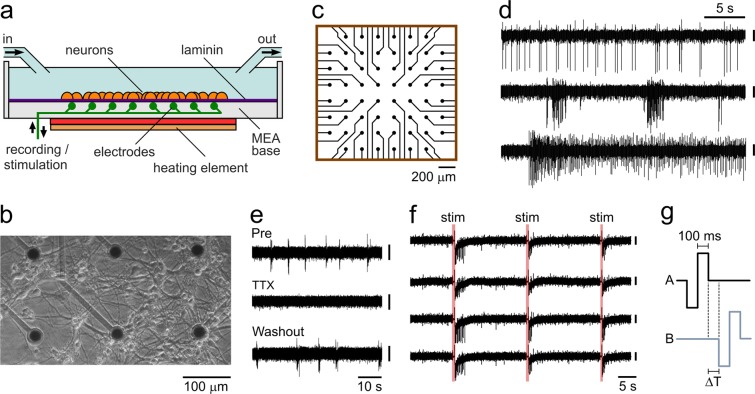
Multielectrode array (MEA) approach. (**a**) Schematic of chamber set-up. (**b**) Representative image of cultured neurons (14 DIV) grown on MEA. (**c**) Detail of 60 electrode array. (**d**) Single electrode recordings showing examples of different firing patterns. Scale bars, 30 μV. (**e**) Sensitivity of spike activity to TTX (500 nM) and washout. Scale bars, 30 μV. (**f**) Stimulation evoked responses in 4 channels. Scale bars, 30 μV, (**g**) stimulus patterns for A and B with training interval delay in between (ΔT).

To investigate the intrinsic mechanisms that contribute to learning temporal relationships in mammalian CNS circuits, we cultured primary hippocampal neurons onto MEA chambers with 60 extracellular electrodes arranged in an 8 × 8 two-dimensional grid pattern (Fig. 1a–c). MEAs allow for simultaneous recording across a cultured network but also provide the means to selectively target stimulation to defined areas of the chamber. Neuronal cultures were plated directly onto the surface of the MEA chamber. To do this, chambers were first sterilized in ethanol, coated with poly-d-lysine (Gibco) and incubated for 4–24 hrs. A 50 μl drop of 4 mg/ml laminin was placed directly over the centre of each array and incubated overnight to provide a focal substrate for cell adhesion. A 40 μl suspension of dissociated hippocampal neurons^13^ from P0 Sprague-Dawley rat pups (sex unknown) was applied to the array centre and neurons were left for 30 mins to adhere before 2 ml of neurobasal A culturing media (Gibco, with 45% glucose and B27/Glutamax supplement) was added to each chamber. Media was supplemented with 4 μM *β*-d-arabinofuranoside (ara-c, Sigma) 48 hrs after plating to prevent excessive accumulation of astrocytes. 50% of the media was refreshed at 7 d after plating, and then at 3–4 d intervals. All experiments were in accordance with the UK Animal (Scientific Procedures) Act 1986 and satisfied local institutional regulations at the University of Sussex, being approved by The University of Sussex Sciences and Technology Cross-Schools Research Ethics Committee.

A 60-channel Multi-Channel Systems MEA system was used for recording extracellular activity. Each electrode was 30 μm in diameter and arranged in an 8 × 8 grid with an inter-electrode distance of 200 μm. For experiments, cultures were recorded in HEPES-buffered extracellular solution containing (in mM): 137 NaCl, 5 KCl, 2.5 CaCl_2_, 1 MgCl_2_, 10 D-glucose, 5 HEPES without synaptic transmission blockers. All experiments were performed at 35 C using an inline heater attached to the perfusion inflow (PH01, Multi-Channel Systems), a heated base-plate and a temperature controller (TC02, Multi-Channel Systems). Cultures transferred to extracellular recording solution were left to settle for 30 mins to equilibrate before each experiment.

**Figure 2:**
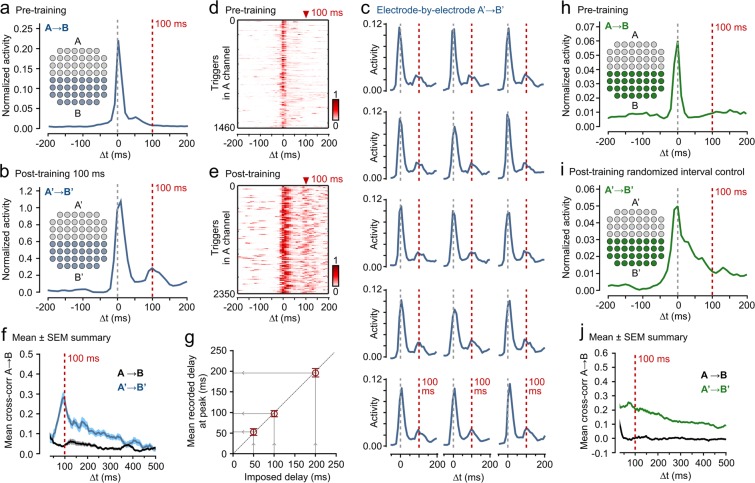
Encoding defined spatio-temporal intervals in the firing relationships between two neuronal populations. (**a**) Pre-training A → B firing relationship. Electrodes were divided into A and B halves (inset) and spontaneous activity recorded for 5 mins. Plot shows example mean A → B response profile. (**b**) A’ → B’ (post-training) response for same electrodes after 100 ms training protocol with additional peak at trained interval. (**c**) Responses of individual B’ channels to activity in A’. (**d,e**) Raster plots for pre (**d**) and post-training (**e**) for experiment from (**a,b**). (**f**) Mean A → B (pre, black) and A’ → B’ (post, blue) cross-correlation plots for 100 ms training experiments (n = 12). Shaded regions are SEMs. (**g**) Summary scatter plot showing imposed delay in training protocol (50 ms, n = 10; 100 ms, n = 12; 200 ms, n = 6) against mean recorded delay. Error bars/shaded region show SEM. (**h**) Encoding control based on randomized A → B training interval. Plot shows example mean A → B response profile. (**i**) A’ → B’ (post-training) response profile for same electrodes at 1 h after protocol based on randomized A → B intervals (>−180 to <=180 ms). (**j**) Mean A → B (pre, black) and A’ → B’ (post, green) cross-correlation plots for randomized training (n = 6). Shaded regions are SEMs. Post-training responses are characterized by higher generalized A’ → B’ correlation without defined peaks.

Cultures were used for experiments at 14–21 days *in vitro* when they had formed highly interconnected circuits (Fig. 1b) which generated significant spontaneous spike activity in MEA recording channels (Fig. 1d). As expected, this spike activity was TTX-sensitive (Fig. 1e) and evoked stimulation led to robust spiking (Fig. 1f). To probe the capability of these neural circuits to encode, process and store temporal information, we devised a training approach based on dividing the electrodes in the array into two subsets, referred to as ‘A’ and ‘B’ (Fig. 2a); as detailed later, different spatial arrangements of A and B were used in different sets of experiments. We first recorded spontaneous activity in each culture for 5 mins (sampling rate 10 kHz), and discontinued experiments where the network already exhibited a strong intrinsic A → B temporal relationship. The training protocol consisted of bipolar 1 V stimulations applied to A followed by B channels with a fixed training delay (ΔT:50–200 ms, depending on experiment) where the delay was between the end of the stimulation to A and the start of the stimulation to B, an arrangement that is similar to bursting (Fig. 1g). Stimulation lasted 200 ms (100 ms hyperpolarisation followed by 100 ms depolarisation – MEA stimulations require matched depolarisation to hyperpolarisation). This pairing protocol was repeated every 10 seconds for 1 h (360 pairings). 1 h after the end of this period, spontaneous activity was recorded for 5 mins (sampling rate 10 kHz) and A → B relationships examined (referred to as A’ → B’ to distinguish post-training from pre-training). This protocol was settled on after it provided reliable interval encoding in preliminary experiments; these preliminary investigations showed that a 200 ms total bipolar stimulation length was the shortest period which gave robust results for a range of delay values, hence it was used thereafter.

### Results and analysis

The first set of experiments divided the electrodes into two spatially defined populations (A,B) which each occupied half the array and were adjoined with a common boundary (Fig. 2a). The training protocol described in the previous section was applied in multiple experiments for each of three different time delay intervals (50 ms, 100 ms, 200 ms) as well as for a set of controls with randomized intervals. Results are summarised in Fig. 2. The plots shown in Fig. 2a–f refer to a set of 12 experiments where the training interval was ΔT = 100 ms.

Two main levels of analysis were applied to the electrode data. The first, used to create response profiles as described next, involved filtering and pre-processing of the data, but is very helpful for visually illustrating overall trends, and is the method employed in the proprietary MC_rack software to visualise relationships between MEA channels. The second – cross-correlation analysis, as explained later in this section – did not employ any pre-processing or simplifications of the data, basing its calculations on the original fine timescale electrode data. This second quantitative method should be regarded as more accurate and is the source of all our main conclusions.

To give an initial assessment of the distribution of time differences between spikes in active B electrodes in response to spikes in A, we generated response profiles. Figure 2a shows a typical firing response profile for an experiment pre-training (A → B), while Fig. 2b shows the same experiment post-training (A’ → B’). These response profiles represent the mean firing temporal relationships between the two populations of electrodes.

Following the method provided in the MEA system proprietary software (MC_rack), response profile histograms were produced by calculating time differences between individual spike events in the ith A channel, a_i_, (a_i_^n^) and all spikes across all B electrodes for 500 ms before and after the event, producing a 1 s window of responses centred on a_i_^n^. Responses within the 1 s window were aggregated into 10 ms bins to give a distribution of spike time delays. When there are multiple sequential spikes in A and B, it can be difficult to discern the temporal relationship from histograms from all-to-all differences as a spike in B can contribute different delays from bursts of spikes in A. In our more detailed analysis, we thus use correlational and causal measures to assess the actual temporal relationship (see below). However for graphical purposes, it is more informative to show histograms from samples of spikes in A and in which a spike in B reports only one delay in the histogram. Following the procedure of the MC_rack software, to make sure that a spike event in B is only counted once in these histograms, responses were collated for a spike, a_i_^n^, and then any further spikes in a_i_ discounted for the next 1 s. In this way, the 1 s window starting 500 ms before the next spike did not overlap with the current window (which ends 500 ms after the current spike).

These response profiles, when summed across all the active B electrodes over the full duration of the experiment, revealed the response to individual spikes in a_i_ and were used to generate each row in the raster plots shown in Fig. 2d,e. Total response distributions were calculated by summing the binned data from each of the selected spikes in a_i_ and normalized by dividing by the total number of responses. Mean responses for an experiment were generated by averaging the normalized response histograms from all the a_i_, thereby giving equal weight to all electrodes. We discounted any that had too little activity to give a meaningful distribution (<50 spike events over the whole 5 min recording) – in practice only a tiny fraction, less than 2%, of electrode recordings fell into this category; while including them did not affect overall results significantly, the 50 spike threshold was chosen to reduce undue influence from these noisy distributions. These mean response histograms are shown in Fig. 2a,b.

Both pre and post-training A → B mean response profiles revealed a discrete peak at delay (Δt) = 0 ms, corresponding to synchronous activity in the highly interconnected network (Fig. 2a,b) but, significantly, the post-training relationship (A’ → B’) was also characterized by a robust peak centred at delay (Δt) = 100 ms (Fig. 2b). We also observed this specific interval at the level of individual electrode activity (each B electrode triggered by A activity, Fig. 2c), implying that it was a preserved feature in the network as a whole. A defined vertical band in single post-training A’ → B’ raster plots demonstrated that the Δt = 100 ms interval also persisted over the recording duration (Fig. 2d,e).

While the response histograms, raster plots and per electrode activity plots produced using MC_rack (Fig. 2a–e,h,i) give a very useful illustration of trends, and provide an intuitive visual picture of the interval encoded, the temporal binning and processing of data inherent in the method makes them a slightly coarse analysis tool since they necessarily rely on a sample of spikes in A. Hence cross-correlation analysis was used to better quantify our findings. This analysis was carried out with our own software which did not inherit any of the constraints of MC_rack. Cross-correlation analysis used all of the data, without any of the pre-processing and filtering required for the response histograms, it should therefore be regarded as more accurate. We generated an average cross-correlation measure for all A → B pairs for each experiment using complete sets of spike train data from each electrode. A summary plot shows the mean ± SEM of these average plots both pre and post-training for all experiments (n = 12, Fig. 2f). As expected, the pre-training relationship revealed a significant peak at zero delay corresponding to synchronous activity in the network, but no other correlations (Fig. 2f, black trace, zero delay not shown as plot starts at 20 ms to make later features clearer). By contrast, the post-training cross-correlation profile (Fig. 2f, blue trace) exhibits a strong peak centred around delay (Δt) = 100 ms (97.6 ± 6.7 ms) consistent with a faithful encoding of the imposed 100 ms training interval across experiments.

Our cross-correlation analysis also allowed us to study the temporal properties of the data at fine timescales based on all spikes recorded, since this method is not sensitive to the potential double-counting inherent in generating response frequencies using the coarse binning method discussed earlier in this section. The normalised cross-correlation between two signals S_1_ and S_2_ (in this case spike trains recorded at pairs of MEA electrodes) was calculated as:

1 $$C(\tau )=\frac{1}{\sqrt{{S}_{1}^{0}{S}_{2}^{0}}}\mathop{\sum }\limits_{t=0}^{N-\tau }{S}_{1}(t){S}_{2}(t+\tau )$$

where *τ* is a delay, or lag, N is the duration of the signals (in this case at least 5 mins), and S^0^ is the autocorrelation of the signal at zero time lag (cross correlation with itself)^14^. Investigation of the autocorrelation properties of the individual signals (pre and post) using Lyjung-Box Q tests revealed that typically there was no significant autocorrelation structure in the signals. However, occasionally there was some marginally significant autocorrelation structure present, so to avoid any possibly spurious results, a corrected cross-correlation calculation, *C*_*c*_, was used throughout. This involved subtracting the cross-correlation calculated with a randomly shuffled version of *S*_1_ which removes correlations arising purely from inherent auto-structure in the signals^15^. The calculation was as follows:

2 $${C}_{c}(\tau )=\frac{1}{\sqrt{{S}_{1}^{0}{S}_{2}^{0}}}\mathop{\sum }\limits_{t=0}^{N-\tau }{S}_{1}(t){S}_{2}(t+\tau )-\frac{1}{\sqrt{{S}_{{1}_{ran}}^{0}{S}_{2}^{0}}}\mathop{\sum }\limits_{t=0}^{N-\tau }{S}_{{1}_{ran}}(t){S}_{2}(t+\tau )$$

where ${S}_{{1}_{ran}}$ is a randomized version of ${S}_{1}$with no temporal structure.

The time step used in the summation was 0.1 ms. Cross-correlation sequences were produced by calculating the cross-correlation at all delays from 0–500 ms in steps of 0.1 ms (i.e. at 5000 different delay values). In order to identify changes in behaviour after training, for all experiments the pre-training cross-correlation sequences (describing spontaneous network behaviour) were subtracted from the post-training sequences. It is these modified post-training sequences (*C*_*c*_^*mod*^ = *C*_*c*_^*post*^ *− C*_*c*_^*pre*^) that are shown in all the figures; pre-training sequences are unmodified. In most cases there is little essential difference between the raw post-training sequences and the modified ones as the pre-training sequences are generally flat and numerically small. However, this modification highlights post-training differences in the cases where there are more noticeable correlations present in the pre-training data. Cross-correlation calculations were not reliable in cases where neuronal activity in recorded datasets was too low, so datasets were not used if more than 50% of active channels had <500 events in a 5 min period (amounting to activity on less than 0.016% of recorded samples). In practice this condition was very rare (and may well have been due to faulty MEA dishes) so less than 1% of data was rejected.

Cross-correlation sequences were calculated for all possible (A,B) electrode pairs during an experiment and then averaged into a single sequence. Such sequences were averaged across all experiments in a set to produce the final mean cross-correlation plots as shown in Fig. 2f, for example.

Significantly, the results found for the 100 ms delay experiments generalised: training to different interval delays yielded similarly matched relationships (Fig. 2g, imposed: 50 ms, recorded: 52.8 ± 8.1 ms, n = 10; imposed: 200 ms, recorded: 196.2 ± 10.1 ms, n = 6). The average recorded temporal relationships (interval encodings) were determined from the average position of the dominant peak in the cross-correlation sequences generated for each experiment in a set.

A set of control experiments with randomised training intervals failed to produce any A’ → B’ relationships. In these experiments control networks received equal stimulation to those in the other experiments but the A → B interval training lag was randomized. On each cycle of an experiment the delay value was drawn randomly from the integers r, where −180 < r < = 180, without replacement, to ensure a uniform distribution of all possible relationships in the interval (−180 ms,180 ms]. These controls yielded a generalized post-training increase in activity, but no structure or peak was observed in the response profiles or mean cross-correlation sequences (Fig. 2h–j). In other words, since no specific, fixed temporal relationship was present during training, none was learnt.

Taken together, our findings demonstrate that even in small reconstituted *in vitro* networks, an imposed interval can be readily and stably stored in the temporal architecture of firing relationships between neuronal populations.

## Generalized Storage of Trained Intervals Across Circuits

### Materials and methods

**Figure 3:**
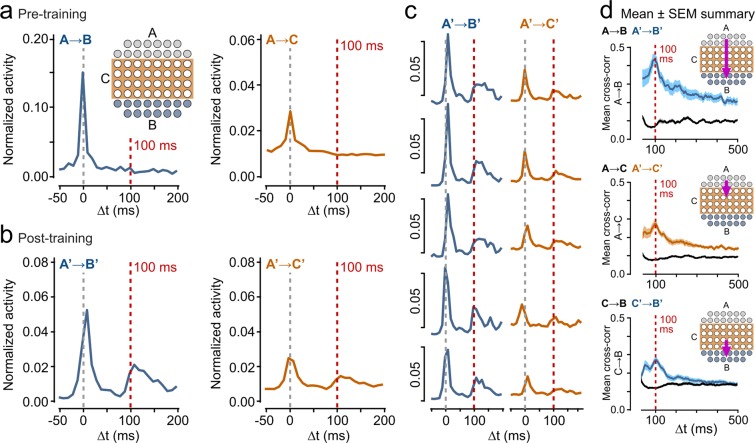
A → B interval encoding across an untrained partition. **(a)** Schematic illustrates training arrangement with A and B nodes separated by a partition of electrodes that were not stimulated ‘C’ (orange). Plots show example mean A → B and A → C cross-correlograms. **(b)** A’ → B’ and A’ → C’ post-training response profiles for same electrodes after 100 ms training protocol. **(c)** Example responses of individual B’ channels to activity in A’ (left) and individual C’ channels to activity in A’ (right). **(d)** Mean cross-correlation plots for 100 ms training experiments (n = 12) for A → B, A → C and C → B nodes pre- and post-training. Shaded regions are SEMs.

To characterize the robustness of the encoding mechanism, we next examined whether we could successfully train A → B when the A and B regions were physically separated by a partition region (C) where electrodes were not stimulated (Fig. 3a), an arguably more realistic analog of input organization in native cortical structures. We reasoned that this arrangement might reveal additional spatial features of encoding in the non-stimulated C region which effectively acts as a conduit for information flow^16^.

In this case, the same experimental method and training protocol was used as for the earlier set of experiments described in the previous section. The relative sizes of regions A, B and C, and the number of electrodes contained in each, are as shown in Fig. 3. Clearly there are now fewer electrodes in both A and B than in the earlier experiments.

### Results and analysis

In a set of 12 experiments we found that using this paradigm with a training interval ΔT = 100 ms, A’ → B’ intervals were still successfully encoded with a dominant peak present at ~100 ms in both the average (Fig. 3b) and electrode-by-electrode post-training relationships (Fig. 3c), and in the mean cross-correlation profile (Fig. 3d, left panel, n = 12). The response profile and cross-correlation analyses used the same methods as described in the previous section .

It is notable that this paradigm gives an even more defined A’ → B’ temporal encoding outcome than the earlier non-separated paradigm (significantly higher mean 100 ms cross-correlation peak height for separated: 0.41 ± 0.03, n = 12, Fig. 3d, compared to non-separated: 0.28 ± 0.02, n = 12, Fig. 2i, p = 0.0031, Wilcoxon rank sum test) suggesting that the presence of intermediate pathways improves the effectiveness of encoding. Notably, A’ → C’ and C’ → B’ relationships also revealed 100 ms peaks (Fig. 3a–c, orange traces), although these were significantly lower than A’ → B’ for the cross-correlation peaks in Fig. 3d (A’ → C’ versus A’ → B’: p = 0.0041, n = 12; C’ → B’ versus A’ → B’: p = 0.0038, n = 12, Wilcoxon rank sum test) and less defined, being both broader and with evidence for additional dominant peaks at shorter intervals (A’ → C’ and C’ → B’ mean peak region full width measured at half the maximum height: 105 ms, A’ → B’: 55 ms; A’ → C’ and C’ → B’ mean peak height: 0.22 ± 0.02, A’ → B’ mean peak height: 0.41 ± 0.03, n = 12, Fig. 3d). This suggests that while the differences in expression are not consistent with a simple uniform whole network temporal response, some trace of interval encoding is captured across the array, presumably through the development of functional A’ → C’ → B’ pathways.

**Figure 4:**
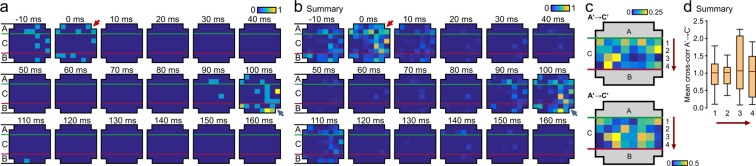
Spatial characteristics of temporal encoding. (**a**) Color-coded raster map of electrode activity in a post-trained network aggregated within electrodes across 10 ms windows before/after a single spontaneous spike in A (red arrow at 0 ms, range of interval windows examined: Δt: −10 to + 160 ms). The trained interval appears as a secondary bout of activity in B at Δt: 100 ms (blue arrow). **(b)** Summary for readout as above across 5 mins of spontaneous activity for a single culture. Responses are summed over spikes in A in which the spike count across active B peaks at Δt = 100 ms (n = 229 responses) ignoring the first 50 ms post firing. **(c)** Heatmaps showing the average A’ → C’ cross-correlation at a lag of 100 ms for two representative 100 ms delay experiments. For each C the value is averaged across all A’s. **(d)** Average A’ → C’ cross-correlation for each row (1–4) in the C region, ordered by distance from the A region, averaged across all experiments (n = 12). Boxes show medians (red lines) and quartile range; whiskers show full range.

To look in more detail at the spatial pattern of activity across electrodes in response to individual spikes in A, we examined the number of spikes at each electrode resulting from a single spike in one of the A electrodes (Fig. 4a,b red arrows) during 10 ms bins, from 10 ms before, to 160 ms after, the triggering spike. The number of spikes generated at each electrode are directly represented by colour-coded cells in the map at the positions of the A, B and C electrodes on the MEA grid. The expression of the stored interval manifests itself as a secondary bout of activity in B at Δt = 100 ms (Fig. 4a,b, blue arrows) that follows a period of quiescence, with activity also apparent in C at the same interval. This is also seen in the average response (Fig. 4b) across spikes in that same electrode and experiment that generated a delay of 100 ms (i.e. spikes that resulted in a response histogram with a peak response in the 100 ms bin, ignoring the first 50 ms after firing to avoid synchronous activity dominating). This analysis shows both that the 100 ms delay is not caused by continuous chains of activity throughout the MEA, nor that it is simply the whole MEA array ‘beating’ at 10 Hz (as there is less activity in A at 100 ms than there is at 0, especially for the triggering spike (Fig. 4a).

While the analyses shown in Figs. 3d and 4a,b demonstrates a trace of the learned interval in C, we reasoned that the encoding might not be homogeneous across the C region, instead perhaps being expressed in a gradient of encoding effectiveness across rows spatially distributed between A to B (increasing with proximity to B). To examine this idea across a set of 100 ms delay experiments, we generated a heatmap for each experiment showing the average cross-correlation at delay Δt = 100 ms from all A electrodes to a given C electrode, mapped directly onto the spatial arrangement of the electrodes. In each case, different and highly non-uniform heatmaps were generated, likely reflecting differences in the spatial architecture of neurons in the necessarily different cultured networks used in each experiment (Fig. 4c). However, when averaged across all experiments, no clear spatial gradient of A’ → C’ → B’ encoding was observed (one-way Kruskal-Wallis ANOVA, p = 0.8466, not significant, 4 groups each with n = 12, Fig. 4d), further suggesting that the learned trace was generalized in the network, but in such a way that the required A’ → B’ response is the strongest characteristic of the network activity.

Overall, our findings demonstrate that spatial properties of learnt temporal relationships can be faithfully preserved in the network architecture even when conveyed through untrained intermediate pathways.

## Ruling Out Simple Synchronisation and Temporal Correlation

Although unlikely – given the randomised training interval controls and the apparently successful encoding of multiple different time intervals (Fig. 2) and the spatial pattern of activity in Fig. 4 – it was considered possible that our observations were caused by spurious synchronisation and temporal correlation effects stemming from the repeated regular stimulation of the A and B electrodes, and did not in fact represent the encoding of a A’ → B’ temporal relationship. It is known that temporal correlation and coupling can sometimes occur in the nervous system without necessarily requiring direct synaptic connections, for instance when separate areas receive correlated external input^17^. Hence additional analysis was performed on the experimental data to help rule this out for both the spatially separated (A,C,B) and non-separated (A,B) scenarios.

This time, instead of analysing correlations between As and Bs from the same experiment, and hence the same MEA chamber – where a cultured network connected the regions – the As and Bs were taken from consecutive experiments, and hence different chambers. In this case there was no possibility of learning temporal relationships through plastic changes in a network connecting the A and B neural populations. However, the training protocol was identical in each experiment, the period of recording was the same, as was the timing of the first stimulus and the relative timings of all subsequent stimuli, and taking consecutive experiments meant that all other conditions were the same. This meant that if there was some spurious correlation effect driven solely by external stimuli, rather than generated by network activity, then it should show up in cross-correlation analysis of the As and Bs taken from different chambers in this way.

**Figure 5:**
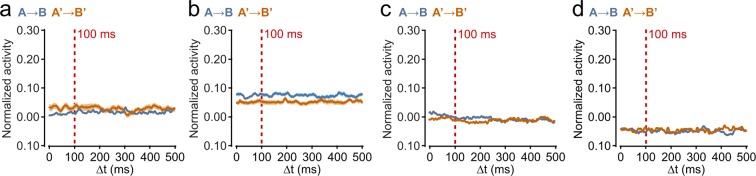
Cross-correlation sequences, A and B in different MEA chambers. (**a**) pre- (A → B) and post- (A’ → B’) training, training interval ΔT = 100 ms, As and Bs each over half of array, n = 12 (cf. Fig. 2f). (**b**) ΔT = 100 ms, As and Bs as in wide separation spatial arrangement, n = 12 (cf. Fig. 3d). (**c**) ΔT = 50 ms, n = 10. (**d**) ΔT = 200 ms, n = 7. Shading shows SEMs (very small in some cases).

The results of this analysis is illustrated in Fig. 5a–d. The cross-correlation sequences were generated as before. In all cases (100 ms training interval on separated and non-separated A and B regions, 50 ms training interval, 200 ms training interval) there was no correlation at all between pre- or post-trained As and Bs. There was also no peak at delay Δt = 0, reflecting the fact that there was no spontaneous synchronous activity as the As and Bs were not part of the same highly interconnected network.

These results add weight to our claim that we are observing the learning of imposed temporal relationships in simple *in vitro* networks and not some spurious correlation effect.

## Information Changes

To test the idea that our training paradigm was associated with a change in information in the network, presumably associated with functional/structural changes, we calculated the level of the information theoretic measure mutual information (MI) between the signals in the A and B populations, before and after training, for the set of 12 experiments referred to in Fig. 3 (training interval ΔT = 100 ms, separation of A and B regions by untrained region C), as well as for the 12 experiments referred to in Fig. 2 (training interval ΔT = 100 ms, A and B regions not separated).

MI is a well-established measure that quantifies how much information in one signal is contained in the other^18^ and we hypothesized that increased MI might accompany training, indicative of information storage, functional changes to the network and possibly suggesting information flow. Consistent with this prediction, change in MI yielded a positive value in all experiments (Table 1). The percentage change in MI was calculated for all (A,B) electrode pairings and then averaged to give a summary value for each experiment.

**Table 1:** Changes in mutual information after training.

Experiment	Average ΔMI (%)	SEM	N	p
A, B separated	266	±111	12	0.023
A, B not separated	215	±96	12	0.021

ΔMI refers to the percentage change in MI after training averaged across all pairs of A,B electrodes; SEM is standard error of the mean, n is the number of experiments in the set, p is the p value for the Wilcoxon rank sum test applied to test the significance of the change in MI between the pre- and post- training populations.

For both sets of experiments, the average increase in MI after training was statistically significant (p < 0.025, Wilcoxon rank sum test, Table 1). Notably, the change in MI was higher in the case where the A and B regions were separated, perhaps related to the observation that the 100 ms cross-correlation peak was also more pronounced for this set of experiments.

The MI between two signals, $I({S}_{1};{S}_{2})$ – in this case spike trains recorded at pairs of MEA electrodes – was computed as follows^19^:

3 $$I({S}_{1};{S}_{2})=\sum _{x\in V{S}_{1}^{\,}}\sum _{y\in V{S}_{2}^{\,}}p(x,y)log(\frac{p(x,y)}{p(x)p(y)})$$

where VS_1_ and VS_2_ are the possible values the signals S_1_ and S_2_ can take respectively (represented by the variables *x* and *y*, in this case each has two possible values: ‘spike’ or ‘no spike’), p(x,y) is the joint probability distribution and p(x), p(y) are the marginal probability distributions. The joint probability distributions were estimated by counting relative frequencies across the entire signal sample at 0.1 ms intervals (amounting to at least 3 × 10^6^ samples for each signal), the marginal distributions were similarly estimated from the empirically observed data contained in the entire signal sampled at 0.1 ms intervals. The increase in MI is probably closely related to the observed general increase in post-training correlation observed across all time delays (including zero lag) in all cross-correlation plots, with larger, peak increases at the imposed training delays, (Figs. 2f,i, 3d). However, since MI is an instantaneous measure, and the time for signals in A to affect B is finite and larger than the time slices used in the MI calculations, because of the spatial separation between the A and B populations (larger in the ‘separated’ experiments (Table 1), but still existent in the ‘non-separated’ experiments), it is not obvious what processes underly the increases in MI after training. One possibility is that the change in mutual information is caused by a stronger dependence of *x* and *y* (Eq. 3) on a hidden variable *z*. A plausible candidate for *z* is the average neuronal firing rate of the whole network, which we had observed tended to increase after training. This was tested by computing the conditional mutual information $CI({S}_{1};{S}_{2}|Z)$, where *Z* represents the average whole network activity (as a spike train) and the variable *z* takes the possible values in *Z* (spike or no spike). *CI* was computed as follows^20,21^:

4 $$CI({S}_{1};{S}_{2}|Z)=\sum _{z\in VZ}\,\sum _{x\in V{S}_{1}}\sum _{y\in V{S}_{2}}p(x,y,z)log(\frac{p(z)p(x,y,z)}{p(x,z)p(y,z)})$$

where *VZ* is the set of possible values *z* can take. The Z spike trains were created in two ways: (i) for each experiment the average network activity was represented by the spike rate per recording time slice averaged over all MEA electrode recordings (separately for the pre and post training conditions), representative spike trains (one for pre, one for post) were then generated using these probabilities with the same sampling rate as for S_1_ and S_2_; (ii) a representative spike train was generated from the spike trains recorded at each electrode by calculating the average time of a spike ‘event’ across all electrodes during a 0.5 ms wide time-window. The window was slid across the entire recording period (again, one for pre and one for post training conditions). The marginal and joint probability distributions were estimated, as for the MI calculations, by counting spikes and spike co-occurrences in the empirical data (using the generated *Z* sequences for calculations involving *z*). These calculations showed that (i) there was a significant increase (p < 0.001, Wilcoxon rank sum test) in whole-network average spiking rate after training for all experiments (average increase for the ‘separated’ set of experiments: 15.4 ± 2.1%, for the non-separated set of experiments: 14.7 ± 2.8%); (ii) conditioning of the mutual information on Z was appreciable (in both pre and post conditions) and the average interaction information ($I({S}_{1};{S}_{2})$- $CI({S}_{1};{S}_{2}|Z)$) across both sets of experiments, in both pre and post conditions, was positive (in fact it was positive for all individual experiments) indicating redundancy among the variables, which is interpreted as meaning that Z accounts for at least some of the correlation between *S*_1_ and *S*_2_^22,23^. Results were very similar for both methods of generating the Z sequences (spiking rates are low so the spike trains are sparse, hence both methods produced similar sequences). The fact that the MI is appreciably conditioned on the average network activity, which increases after training, at least partly explains the increase in MI. There may be conditioning on other hidden variables, or even persistent, spatially extended modulatory processes at play, but investigation of such possibilities would require very extensive further empirical work that is outside the scope of this paper.

Since MI does not explicitly measure directions of information change or flow, and does not take account of temporal structure in the signal, it was merely used as an initial (relatively cheap to calculate) indication that there had been some (functional) changes in the network post-training. Once the MI finding was confirmed, we therefore also examined changes in information flow using the much more expensive transfer entropy (TE) measure, a quantity that identifies the direction of information transfer between two processes^24^ and which is sensitive to temporal structure. It was originally introduced as a value ‘that shares some of the desired properties of mutual information but takes the dynamics of information transport into account’^24^. We applied transfer entropy analysis to identify the direction of information transfer between (A, B) electrode pairs in experiments referred to in Fig. 3 (training interval ΔT = 100 ms, separation of A and B regions by untrained region C). Because the calculations were very computationally expensive and time-consuming, a randomly-selected subset of four experiments were used in preliminary investigations. In these we examined whether TE could reveal information flow from A to B after training. TE from S_1_ to S_2_ is defined as follows^24^:

5 $$T{E}_{{S}_{1}\to {S}_{2}}=\sum _{{S}_{2}^{t},{S}_{1}^{t-1:t-{L}_{1}},{S}_{2}^{t-1:t-{L}_{2}}}p({S}_{2}^{t},{S}_{1}^{t-1:t-{L}_{1}},{S}_{2}^{t-1:t-{L}_{2}})\log (\frac{p({S}_{2}^{t}|{S}_{2}^{t-1:t-{L}_{2}},{S}_{1}^{t-1:t-{L}_{1}})}{p({S}_{2}^{t}|{S}_{2}^{t-1:t-{L}_{2}})})$$

where ${S}_{n}^{t}$ is the value of *S*_*n*_ at time *t*, ${S}_{n}^{t-1:t-{L}_{n}}$ is the (history) sequence of values of *S*_*n*_ over the past *L*_*n*_ timesteps, and the sum is over all possible combinations of ${S}_{2}^{t}$ and the two histories. TE is a measure of conditional mutual information, contingent on the past histories of *S*_1_ and *S*_2_ to *L*_1_ and *L*_2_ previous timesteps respectively. It is closely related to Norbert Wiener’s idea that a source system influences a target system if knowledge about the past of the source improves predictions about the future of the target, beyond predictions based on the past of the target alone^25^.

Assuming stationarity, TE was estimated for pairs of signals (spike traces from MEA electrode readings) by estimating the constituent probabilities in Eq. 5 (after rewriting conditional probabilities in terms of joint and marginal probabilities) by counting occurrences of the relevant patterns over the entire signal sample (as in^26,27^). In order to make the computations tractable, the signals were resampled into bin size 0.5 ms; making the common assumption^27^ that the influence of the target’s own history would quickly reduce, *L*_2_ was set to 5 and *L*_1_, the history of the source, was set as long as possible (=20) without the computations becoming infeasibly time-consuming (the TE calculations scale roughly as ${2}^{({L}_{1}+{L}_{2})}$). We were specifically looking for evidence of information transfer at the imposed training interval, hence counts of joint occurrences of spikes in both processes were separated by a specified delay (100 ms)^26,27^. To help mitigate potential finite sampling inaccuracies, an effective measure, *TE*^*e*^, was used which involved subtracting the TE of a shuffled (randomised) version of the first signal to the unchanged second signal from the TE between the unchanged signals as defined above (i.e. $T{E}^{e}\,=\,T{E}_{{S}_{1}\to {S}_{2}}-T{E}_{{S}_{1}^{^{\prime} }\to {S}_{2}}$, where ${S}_{1}^{^{\prime} }$ is the shuffled version of ${S}_{1}$)^28^.

We tested for information flow (at a 100 ms delay) from A to B by calculating *TE*^*e*^ between all (A, B) electrode pairs in the data subset analysed and then averaging. We calculated a significant increase in average transfer entropy from A to B after training (37 ± 8.4%, n = 4, p = 0.0153, two-sample t-test, test statistic = −4.0442, d.f. = 6) consistent with an increased flow of information from A’ → B’ compared with the baseline, background *TE*^*e*^ of A → B. Conversely, there was no significant increase in *TE*^*e*^ for B’ → A’, leaving us to conclude that training increases *TE*^*e*^ in one direction (from As to Bs) commensurate with information flow occurring in that direction at a delay of 100 ms.

## Encoded Temporal Relationships Can be Copied Into Naïve Networks

We have shown that a specific interval can be stored in the temporal architecture of relationships between two neuronal populations in an inter-connected network. This would have key additional relevance if the encoded information seen in the delayed responses could act as a template to impose changes in the temporal architecture of other independent networks; an *in vitro* analog of a replicative capability that would be of significant interest in the intact nervous system. We designed an experiment to investigate this possibility.

### Materials and methods

**Figure 6:**
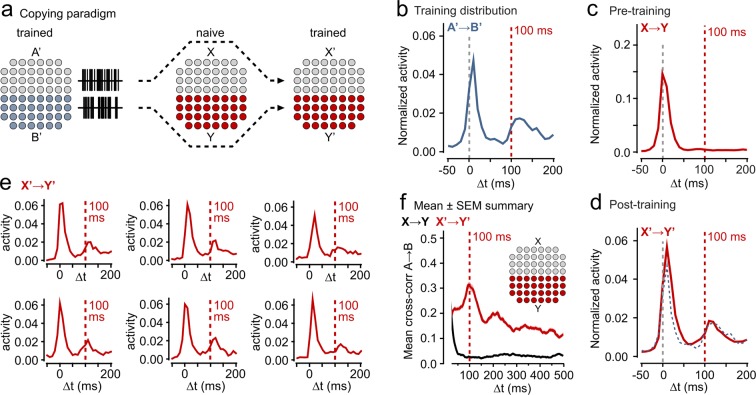
Copying activity relationships between circuits. (**a)** Schematic illustrating experimental scheme for replicating a trained activity relationship in a naïve circuit. Spontaneous output from a culture trained to encode a 100 ms A’ → B’ interval was subsequently imposed on a naïve culture (X, Y). **(b)** A’ → B’ response profiles showing post-training relationship using for copying. **(c)** Plot shows example mean X → Y (pre-training) response profile. **(d)** X’ → Y’ (post-training) response profile for same electrodes after copying protocol. **(e)** Example responses of individual Y’ channels to activity in X’. **(f)** Mean cross-correlation plots for copying (n = 11 experiments). Shaded regions are SEMs.

Specifically, we tested whether an A’ → B’ relationship that was first encoded by our 100 ms training paradigm (see methods in Section ‘Robust Learning of Temporal Relationships in a Simple Network’) could subsequently be used to train the same interval between two neuronal populations (X, Y) in a new naïve neuronal culture (Fig. 6a,b). As previously, we first recorded pre-training spontaneous activity to assess any intrinsic X → Y relationships (Fig. 6c). In the training phase, activity drawn from the output of A was imposed on region X and activity from B onto region Y (Fig. 6a).

Because of fundamental limitations of the MEA system, it was not possible to use the (A’,B’) spike trains directly as training stimulation patterns for (X,Y). Rather, they had to be converted such that training stimuli derived from them, and preserving the most important temporal relationships, could be used within the constraints of the MEA. Essentially, this involved low-pass filtering the activity across A and B, and removing synchronous activity, so that a stimulation pattern could be imposed. As such our copying protocols draw their fundamental timing properties from the original activity but have been inevitably adapted to suit the hardware constraints described below.

One fundamental limitation of the MEA system is that, although individual electrodes can be read at a fine timescale, the same is not true of stimulation. Electrodes cannot be individually and asynchronously stimulated. There are only two stimulation channels which can each be applied simultaneously to populations of electrodes (i.e. all electrodes in the population must be stimulated at the same time): one population can be stimulated simultaneously followed by another population. A second issue is that we cannot apply excessive stimuli to the cultures (e.g. continuously repeated stimulation) without causing damage. Hence the first part of the transformation of the (A’,B’) spike patterns into a training stimulus for (X,Y) is to aggregate the individual spike trains into single patterns (one derived from A’, one from B’) to take into account these two limitations while reflecting significant spiking events across the A or B electrodes. This was achieved by using a variant of burst detection, in which consecutive 40 ms windows were examined and if the total number of spikes across a given population was >6 (20% of the 32 channels) within a time window, we set a merged spike in that window at the average of the spike times. After initial investigation, the 40 ms window length and 20% parameters were chosen so that, in combination with the following steps, the overall number of spikes was similar to the standard training used in earlier experiments (Sects. 2–5) while still being representative of the output.

A third inherent limitation of the MEA system is the requirement of a finite length MEA stimulation period. Hence the next stage of the transformation of (A’,B’) spike patterns to (X,Y) training stimuli was to remove any spikes within the stimulus length (200 ms) of the previous spike to avoid stimulus overlap (as a second stimuli cannot be applied while the first is still underway). This can be done separately for the transformed A’ and B’ patterns, starting with the first spike and iterating forward. However, because of the highly interconnected nature of the networks there is always strong synchronous activity, particularly in trained networks such as (A’,B’), so any stimulus in A’ that generates a delayed response in B’ is also likely to create synchronous activity in B’. If we were to remove all spikes within 200 ms of this kind of synchronous activity in B’ (caused by A’) in order to create the Y training stimuli, it would be impossible to train for any delay outside the near synchronous activity (in the recording we used, this effectively removed all A-B time differences in the range ±[15, 200] ms). Since (A’,B’) was trained for a 100 ms delay this presents a problem as all we would be able to entrain would be the synchronous activity at close to zero delay, or delays >200 ms. Hence in a final step in the transformation to training stimuli we filtered out the synchronous activity, such that we were effectively attempting to copy from (A’,B’) but without synchronous activity. To achieve this, we removed any spikes in B’ <50 ms after the onset of a stimulus in the training protocol for X (derived from A’ following the steps outlined above). This created the final Y stimulus derived from B’. 50 ms was chosen after preliminary investigations as the shortest period that filtered out synchronous activity and allowed temporal correlations in the required range while still being representative of the original spiking patterns. All other experimental methods were as before (see methods in Section ‘Robust Learning of Temporal Relationships in a Simple Network’).

### Results and analysis

The analysis methods were as before (see Section ‘Robust Learning of Temporal Relationships in a Simple Network’). Strikingly, we found that the 100 ms peak observed in the A’ → B’ activity was effectively replicated in the X’ → Y’ post-training plot at the level of an individual experiment (Fig. 6d,e) and in the mean cross-correlation profile for all experiments (imposed: 101.1 ms, recorded: 98.4 ± 12.1 ms, n = 11, Fig. 6f). These findings demonstrate that imposed changes in the temporal architecture characteristics of one neuronal ensemble can act as a substrate for information copying to a second naïve circuit. The original imposed training delay is preserved in the stimuli derived from (A’,B’) and has been replicated in (X’,Y’).

## Discussion

Timing external events is a necessary demand in the nervous system, relevant to tasks including sensory integration and learning^29^. The neurophysiological mechanisms responsible are therefore of significant interest but remain poorly defined. A key question is whether interval-encoding requires dedicated central time-keeping circuits or can instead arise from local plasticity properties intrinsic to the neuronal circuits themselves^30^. In support of the latter, recent *tour de force* studies suggest that richly-interconnected cortical brain slices can exhibit interval encoding^2,3^, in effect a form of temporal pattern completion that anticipates an encoded time interval built into the training regime. Similarly, *in vivo* work in visual cortex has revealed how sub-populations of neurons can exhibit activity at a defined interval after a stimulus, corresponding to the timings used previously in a reward-learning task^31,32^. In the present study, we demonstrate a similar capability in a substantially reduced system comprised of two-dimensional sparse reconstituted cultured hippocampal networks. Using multi-electrode array technology we show that an imposed interval of 50–200 ms can be represented in the temporal architecture of the circuit and preserved over a timescale of an hour. The relative numerical simplicity of our network suggests that timing is therefore not just an emergent property of large, richly-interconnected circuits but an inherent capability arising from the interactions between limited numbers of neurons, suggesting that plastic processes in the wiring of reformed circuitry are sufficient to perform this function. Of course, our training regime, which relies on repeated pairings of imposed activity timing intervals over an hour-long timescale, does not correspond to physiologically-relevant input, and thus our findings are not direct evidence for interval encoding mechanisms that might operate *in vivo*. Nonetheless, they illustrate a capacity for simple local circuits to learn such relationships, adding weight to arguments in favour of intrinsic models of temporal processing in neural systems^30^. Our observation of significant increases in measures of information storage and flow, mutual information and transfer entropy respectively, between A and B post-training, provides additional quantitative support for our findings^33^.

It is notable from our data that intervals could be encoded most effectively when A and B populations were separated by a region that was not directly targeted in the training paradigm. This observation suggests that the temporal encoding develops best when intermediate pathways are available between spatially separated input and response regions, compared to the tightly integrated overlapping networks of the non-separated condition, perhaps because the intermediate conduits are less susceptible to interference within the network and between stimulating signals. This is consistent with previous studies suggesting that such interference is known to result in conflict between competing processes of neural plasticity during learning of temporal patterns^34,35^.

A set of preliminary investigations showed that the length of the time step/bin size used in the cross-correlation and information theoretic calculations had some effect on the results obtained. There was no discernible difference in sizes between 0.1–0.6 ms and very little at 1.0 ms. For step sizes larger than 1 ms, trends gradually became less distinct as the step size increased. This is in keeping with previous observations^26^ that step/bin sizes of 1 ms or less are best for accuracy where the data is available at that resolution (as it was here). Hence we adopted the smallest step size available for the data (0.1 ms) for the cross-correlation and MI calculations. To help make the TE calculation feasible a coarser temporal resolution was used in that case as explained in Section ‘Information Changes’.

Beyond its role in event timing, there is potential relevance for information stored in the temporal architecture of a circuit to act as a possible substrate that could drive changes in the activity timing relationships in other independent networks. The idea of such a replicative capability in the nervous system is attractive^12,36,37^ but so far has limited direct experimental support. By exploiting our reduced circuit set-up, we provide evidence for this capability where trained activity patterns derived from the output of one network could be played back to a second. Of course, this is a simplistic example of information replication, and due to limitations of the MEA, necessitated removal of synchronous activity, but implies that one circuit has the inherent capability to impose temporal structure onto a second. In the intact brain, a number of networks have wiring properties and functional demands that would make them plausible candidates for utilizing such a property, for example, the pathways between medial temporal cortex and neocortex^12^ and in cerebellar motor learning circuits where evidence for a shift in a functional memory trace from cerebellar cortex to vestibular nuclei has been implicated^38^. Information propagation is of general and significant interest^39,40^ and further insight into fundamental mechanisms in reduced systems which enable precise control of input patterns, provides the foundations for studying similar capabilities in complex networks *in vivo*. This kind of replication of information and spatio-temporal relationships could potentially also play an important role in adaptive neural dynamics - it could even form the basis of true evolutionary dynamics, an idea that has been speculated on for some time^37,41^. The results reported in this paper suggest that this form of information processing and copying might be an intrinsic property of neural networks, even in sparse, reduced systems.

The ability of the nervous systems to distil temporal regularities from the sensorium, and to generate and manipulate them internally, represent generic and fundamental abilities. Phenomena such as spike-timing dependent plasticity^42^, synfire chains^43^ and predictive coding via Bayesian inference^44^ all involve manifestations of this powerful capability. Our results provide new evidence that such an ability appears to be an intrinsic property of hippocampal neurons such that it will arise even in extremely sparse reconstituted networks. This ties in with recent findings suggesting that neuronal networks try to form models of sequential inputs by attempting to minimise variational free energy so as to maximise the evidence for their implicit models of stimulation^4^. In one sense, our results can also be viewed as reaffirming the notion of synfire chains in a generalised context by showing that *in vitro* neuronal circuits retain the capacity to encode and re-express temporal patterns of firing.

In summary, our results show that sparse reconstituted cultured hippocampal networks are able to extract and replicate temporal regularities found in external stimuli, strongly suggesting this is an intrinsic property of such networks.

## Data Availability

Datasets generated during and/or analysed during the current study are available from the corresponding author on reasonable request. Data will shortly be made publicly available on institutional and public repositories.
